# Practical Benefits, Challenges, and Recommendations on Social Media Recruitment: Multi-Stakeholder Interview Study

**DOI:** 10.2196/44587

**Published:** 2023-05-22

**Authors:** Nina Goldman, Theresa Willem, Alena Buyx, Bettina M Zimmermann

**Affiliations:** 1 Institute of History and Ethics in Medicine Technical University of Munich Munich Germany; 2 Institute for Molecular Immunology Klinikum Rechts der Isar Technical University of Munich Munich Germany; 3 Department of Science, Technology and Society Technical University of Munich Munich Germany; 4 Institute for Biomedical Ethics University of Basel Basel Switzerland

**Keywords:** social media, recruitment, benefits, challenges, recommendations, medical study, interview, research study, strategy

## Abstract

**Background:**

The increasing use of social media opens new opportunities for recruiting patients for research studies. However, systematic evaluations indicate that the success of social media recruitment in terms of cost-effectiveness and representativeness depends on the type of study and its purpose.

**Objective:**

This study aims to explore the practical benefits and challenges of recruiting study participants with social media in the context of clinical and nonclinical studies and provide a summary of expert advice on how to conduct social media–based recruitment.

**Methods:**

We conducted semistructured interviews with 6 patients with hepatitis B who use social media and 30 experts from the following disciplines: (1) social media researchers or social scientists, (2) practical experts for social media recruitment, (3) legal experts, (4) ethics committee members, and (5) clinical researchers. The interview transcripts were analyzed using thematic analysis.

**Results:**

We found diverging expert opinions regarding the challenges and benefits of social media recruitment for research studies in four domains: (1) resources needed, (2) representativeness, (3) web-based community building, and (4) privacy considerations. Moreover, the interviewed experts provided practical advice on how to promote a research study via social media.

**Conclusions:**

Even though recruitment strategies should always be sensitive to individual study contexts, a multiplatform approach (recruiting via several different social media platforms) with mixed-methods recruitment (web-based and offline recruitment channels) is the most beneficial recruitment strategy for many research studies. The different recruitment methods complement each other and may contribute to improving the reach of the study, the recruitment accrual, and the representativeness of the sample. However, it is important to assess the context- and project-specific appropriateness and usefulness of social media recruitment before designing the recruitment strategy.

## Introduction

Social media has changed the interpersonal communication style of many people and has often become a substitute for face-to-face contact, especially with the proliferation of new communication technologies [1]. Together with the increasing use of social media over time [2], this opens new opportunities for recruiting patients for research studies. These opportunities include a more efficient recruitment accrual, as low recruitment rates are a central barrier to the success of many clinical trials [3,4] and the opportunity for community building [5]. A growing number of studies are published that report on effective social media recruitment. For example, social media recruitment has been successfully used in the fields of medicine [6-8] and the social sciences [9,10]. However, systematic evaluations indicate that the success of social media recruitment in terms of cost-effectiveness and representativeness depends on the type of study and its purpose [11]. Furthermore, a lack of consistent reporting on the details of social media recruitment strategies makes it difficult to assess if social media can improve study participation [12]. Some challenges identified concern fraudulent enrollment in web-based surveys when financial compensation is provided as an incentive for study participation [13]. Therefore, it has been proposed to use browser cookies or allow only 1 survey participation per IP address as a protective measure [8].

The optimal use of social media for recruitment purposes depends, for instance, on the study type and the study team’s existing knowledge of social media recruitment [14]. However, the inconsistent reporting of social media as a recruitment tool for research makes it difficult to assess the benefits and challenges of different study designs [15]. Moreover, a review of the effectiveness of social media recruitment has been inconclusive [12]. The lack of guidelines and inconsistent reporting has led us to the following research question: What benefits and challenges do stakeholders perceive regarding social media–based recruitment for research studies? This paper aims to outline context-sensitive practical considerations on aspects deeming social media recruitment suitable and unsuitable for research studies, with a particular focus on clinical versus nonclinical studies. It further aims to formulate recommendations on how to use social media for recruiting purposes.

These aims are addressed in the context of 2 types of studies: clinical and nonclinical studies. First, clinical studies include preclinical trials that focus on evaluating surgical, medical, or behavioral interventions to find a new treatment that is effective and safe to use. This involves healthy participants or patients, with an indication for a particular disease. The second study type includes nonclinical studies disconnected from medical care, health diagnosis, or treatment. These may include, for instance, web-based surveys, observational longitudinal studies, qualitative interview studies, focus group studies, or cross-sectional studies. Regardless of the type of study, there are general legal frameworks such as the General Data Protection Regulation (GDPR) [16] in the European Union, which must be consulted when collecting and processing personal health data. The United States does not have an equivalent to the GDPR. Instead, there are sector- or state-specific data protection regulations [17]. Moreover, the International Ethical Guidelines for Health-related Research Involving Humans [18] and the Declaration of Helsinki [19] provide guidelines on how to ethically conduct research involving human subjects.

## Methods

### Overview

This study is part of the research consortium TherVacB–A Therapeutic Vaccine to Cure Hepatitis B (work package 6), which aims to assess the ethical, legal, and social aspects of social media recruitment in the context of clinical trials. TherVacB is an EU-funded international research consortium conducting a phase Ib/IIa clinical trial on a therapeutic vaccine against hepatitis B [20]. The TherVacB consortium is—among others through this study—assessing if social media recruitment is an applicable tool for their clinical study. We used the Consolidated Criteria for Reporting Qualitative Research checklist to report on the research team, study design, and data analysis [21].

### Recruitment

Based on a literature review [14], we identified a list of relevant stakeholders to assess the practical, ethical, legal, and social implications of social media recruitment to define the inclusion criteria. Participants who were eligible were researchers or professionals with practical or theoretical knowledge of social media or clinical study recruitment from the clinical, legal, social science, ethics, public relations, and technology domain. The stepwise recruitment process was guided by considerations of theoretical saturation [22]. Initially, potential participants were identified through reference screening and conferences (n=206, 11 participated), existing networks from the TherVacB consortium (n=55, 4 participated), and snowballing (n=16, 3 participated). They were invited via email. After 13 interviews, a preliminary review of the content led us to reassess the stakeholder groups and invite additional legal and ethics experts from Germany, Switzerland, and the United States (n=124, 12 participated) as we found that there were differing opinions between US experts and European experts on data privacy concerns. In addition, we included patients with diagnosed hepatitis B who spoke German or English and were aged at least 18 years as an additional stakeholder group. They were recruited in a clinical setting during regular hepatitis B–related checkups (n=18, 6 participated). Patients with hepatitis B were included in the interview study as they represent a vulnerable patient group, and we wanted to include their perspectives on potentially being recruited via social media. All participants were informed orally and in writing, with a study information leaflet about the content and scope of the study and signed an informed consent form prior to the interview. Participation was not compensated.

### Ethics Approval

The study was approved by Technical University Munich’s ethics committee (431/20 S-KH).

### Data Collection

We conducted semistructured interviews with participants from the following areas of expertise: (1) social media researchers or social scientists that have worked with social media (recruitment), (2) practical experts for social media recruitment, (3) legal experts, (4) ethics committees members from Germany, Switzerland, and the United States, (5) members of the TherVacB consortium, and (6) patients, who use social media or have been in touch with social media recruitment. We used separate interview guides for patients and experts (see Multimedia Appendix 1). The expert interview guide included questions about the ethical, legal, social, and practical aspects of social media recruitment. This guide was then pilot tested with 2 members of the TherVacB consortium and adapted. The pilots were included in the interviews. The patient interview guide included fewer but more open questions targeted at a narrative of experience related to social media use, study recruitment, and lived social experiences with hepatitis B. The patient guide was also tested on 1 patient and refined. Three pilot interviews were included in the analysis. The interviews were conducted between August 2020 and September 2021 by BMZ and TW. The interviewees and the interviewers did not know each other prior to the interview and were only in contact via email (for experts) or phone (for patients). Due to the COVID-19 pandemic, all interviews were conducted on the internet or by phone call. At the beginning of the interview, researchers summarized the aim of the interview and the study and allowed the participants to clarify any remaining questions. Demographic information was gathered at the end of the interviews (if applicable). Each interview lasted between 25 and 60 minutes, was conducted by individual researchers, audiorecorded, and transcribed verbatim either manually or with the assistance of a transcription software, depending on the recording quality (TW, BMZ, and student assistant). Transcripts were pseudonymized by replacing all identifying information with a placeholder. The transcripts were provided to the participants upon their request.

### Data Analysis

The interview transcripts were analyzed using thematic analysis [23] and facilitated by qualitative data analysis software (Atlas.ti version 8.0; Atlas.ti Scientific Software Development GmbH). An initial coding scheme was developed inductively and applied to a subset of interview transcripts (TW and BMZ). The coding scheme was refined subsequently based on regular discussions among the research team (see Multimedia Appendix 1 for the final list of codes). All interview transcripts were then coded according to the new coding scheme and validated by a second coder (NG, TW, and BMZ). Then, themes and categories were created by grouping and relating codes. Memos helped to capture potential analysis angles and link different interviews (NG, TW, and BMZ). After reviewing all codes, we wrote analytical memos to summarize the interview content related to each code (NG, TW, and BMZ). These memos were discussed and related to existing literature. Based on these discussions as well as original quotes from the interviews, the first author (NG) drafted the analytic report for this paper. The report was critically reviewed by all coauthors to refine the analysis (TW, AB, and BMZ).

## Results

### Sample

The participants in our sample have diverse areas of expertise: clinical research (n=10), communication (ie, public relations; n=2), ethics (n=10), law (n=3), philosophy (n=1), psychology (n=1), and social science (n=3); we also included patients with hepatitis B (n=6). Participants spanned the following geographic regions: Germany (n=22), Switzerland (n=3), Spain (n=1), the United States (n=7), Canada (n=1), and Australia (n=2).

There were considerable differences between participants concerning their practical experience with social media recruitment: 13 experts had been directly involved in studies recruiting participants via social media, 17 experts came in touch with social media recruitment by approving studies as institutional review board members or had second-hand or theoretical expertise, and 6 patients with hepatitis B shared their views on the prospect of being recruited via social media. Some experts working in institutional review boards and other ethics committees had observed an increase in the number of studies that plan to recruit via social media. During the interviews and data analysis, we used an inductive definition of social media as provided by the study participants, who perceived social media as communication platforms with large user numbers (Facebook, Twitter, and Instagram were most commonly mentioned) or messenger services (WhatsApp and Facebook messenger were mentioned).

The practical benefits and challenges were perceived differently, even opposingly, among the study participants. We inductively derived four themes where participants expressed opposing views on the benefits and challenges of recruiting through social media: (1) resources needed, (2) representativeness, (3) web-based community building, and (4) privacy considerations. Table 1 summarizes these themes and the main arguments within each.

**Table 1:** Overview of themes demonstrating diverging expert opinions regarding social media recruitment.

Theme	Benefits (+)	Challenges (−)
Resources needed to conduct social media recruitment	Cost-effective and rapid recruitment, especially for large samples Effective in low population density settings and over a large geographical area Easy to conduct and can be done in-house	Does not lead to cost savings as a full-time employee may need to be hired to manage social media recruitment Setting up and implementing a social media recruitment strategy is very time-consuming. Compared to onsite recruitment, social media recruitment requires additional financial and human resources
Representativeness of social media recruitment	Easier to target specific populations For very specific target group, it is more important to reach them at all rather than trying to get a representative sample Reach people you would not otherwise reach as some are only available on the internet Good representation of older and younger adults	Not representative of the study population, as certain demographic groups are overrepresented, while others are underrepresented, especially if the study requires a high number of participants Selection bias is a risk for any study
Web-based community building as part of social media recruitment	Direct contact between researcher and participant is highly valued by participants The possibility to stay in contact with the participants is good for follow-up studies and participant retention Interaction between participants gives them a sense of security and reassurance, and they feel less alone with their disease Enables more targeted recruitment efforts Participants disclose more web-based information than in a face-to-face conversation	Building a web-based community for the participants is associated with higher costs Unblinding of participants may have a negative impact on the quality of the study Social pressure to participate in the study if exerted by personal contacts or within the community Personal contact with the doctor is very important for building trust, especially in the clinical setting
Privacy considerations regarding social media recruitment	The use of social media should be considered a risk of daily life No privacy issues if: advertising on social media does not involve coercion users provide the information intentionally or voluntarily	Particular risks in recruiting via social media The problem of inferential targeting and user profiling through artificial intelligence algorithms used by social media companies leads to privacy violations Uncertainty about what can be done in terms of data protection

### Theme 1: Resources Needed

#### Low Investment

Seven experts recruiting for nonclinical studies considered social media to be a cost-effective and cheap recruitment option especially compared to other forms of advertising. Other experts in psychology and communication who have recruited for cross-sectional and clinical studies were convinced that being able to recruit large samples fast could also help reduce recruitment costs per participant. An ethicist with web-based recruiting experience also noted that social media could reduce the cost of recruitment when it came to reaching out to the general population and conducting opinion polls, as recruitment could easily be done in-house instead of outsourcing it to an external service provider. One of the interviewees worked as an external service provider and agreed that social media recruitment for clinical trials is cost-saving, which this example shows:

I’m just convinced of [cost savings], because the costs per recruitment, so to speak per included patient have partly gone down from 200 to 300 Euros to 20 to 15, 16 Euros. That means that the CROs [Contract Research Organizations] save money with it, of course. Sure.

Communication expert

Two experts with experience in social media recruitment said that advertising their studies on social media had increased their geographical reach and allowed them to reach areas with low population density, for example, Australia. Additionally, social media was considered suitable for recruiting healthy people.

#### High Investment

Some clinical researchers disagreed with the cost-saving rationale regarding social media recruitment. For example, a clinical researcher thought that social media recruitment, compared to venue-based recruitment, was resource intense because it would require additional steps, such as a contact person who would verify the patients and refer them to a doctor or study nurse. In particular, the necessity of well-trained personnel was stressed by another clinical researcher:

But I think for most of these teams, they don’t have the bandwidth and the strategy to really build a community around a certain project, a social media presence, let’s say, on Facebook.... But most teams/it’s just not part of their strategy, not part of their thinking. And I think it’s just, it’s a time issue, right. Because it takes time to build these things and people don’t have the time.

Clinical researcher

A third clinical researcher suggested employing team members with experience in social media who could be responsible for the recruitment. This becomes especially relevant when considering the extensive practical advice laid out in Textbox 1. The necessity of well-trained personnel, therefore, increases personnel costs. Two clinical researchers stated that snowballing or other forms of recruitment were more cost-effective than social media recruitment. Moreover, 3 clinical researchers stated that the recruitment of patients via social media might overload a study’s resource capacity if too many (unsuitable) patients expressed interest in participating, which also increases staff costs. An expert in philosophy stated that it was important to consider that the cost-saving rationale of using social media to recruit patients would not guarantee a qualitative improvement.

**Practical recommendations from the interviewed experts regarding advertising a research study on social media. Unless stated otherwise, recommendations stand for both clinical and nonclinical studies.**

**Preparation**

- Set the budget for recruitment. This includes a review of available internal resources (time, experience with social media recruitment).
- Set the recruitment target.
- Conduct a cultural analysis to write messages that are tailored to the target population. This will help you avoid people feeling offended or excluded by your content (images and texts).
- Avoid stereotyping text and images (eg, a handsome shirtless man for a sexual health study).
- Create a list of organizational pages or areas of interest that your target audience might follow or like.
- Create more content (message or image) than necessary and have everything approved by an ethics committee and institutional review board as some content might be rejected by the social media platform. Then test different promotional images from a library of at least 12.
- Start a project page, publish posts about the study on the page, and create advertisements to drive traffic to the external study website.
- Plan how to respond appropriately to potentially harmful and negative comments and when to delete them, and designate a responsible person for this.
- In the European Union, you need to consider how you will obtain consent to contact the target audience (General Data Protection Regulation).
- Plan how you will check basic eligibility to avoid wasting resources and giving patients false expectations.
- Work with agencies that specialize in social media recruitment if funding is available.

**For clinical studies**

- Disable comments on social media pages to prevent users from posting health information and self-identifying as a person with a particular disease.
- Centralize the execution of the communication strategy in multicenter studies.
- Ensure that direct contact with patients is established at the recruitment stage, as this is essential for building trust.

**Web-based message**

- The message should clearly outline the aims of the study and highlight the benefits of the study.
- Ensure that therapeutic misunderstandings are cleared up at an early stage.
- Explain the inclusion and exclusion criteria as well as possible.
- The message should be a confidence-building call to action.
- Avoid making people from vulnerable populations feel guilty.
- Be transparent about the uncertainty of what social media data will be used for by including a disclaimer in your message. Researchers should protect patients from disclosing their health information on social media, especially if it is a stigmatized disease.
- Provide an email address where participants can contact the study coordinator outside of social media.
- Provide privacy statements to alert recipients to potential privacy risks when using social media.

**Increasing the reach**

- Use several platforms to have a high reach and try to gain followers (if you have a project page or account) to further increase the reach.
- Use community management to make recruitment more specific to the target population.
- If you want to recruit from closed groups (eg, disease-specific groups), contact the moderator first for approval to approach users. These groups are also less harmful in case participants spontaneously reply as it is more private.
- Increase the visibility of the message by packaging the content in a way that many people engage (eg, through likes). This will make the message pop up on their feed for peers to see.
- Regular media exposure, for example, through radio interviews, TV, and media launches helps to increase uptake.
- Avoid using the network of others for recruitment because it is highly problematic.
- Do not overadvertise the study.

**Vulnerable populations**

Experts perceived study participants as vulnerable if they exhibited one or more of the following characteristics: having a stigmatized disease (eg, hepatitis B, HIV, and mental illness), being severely ill (eg, cancer), being a child, living in poverty or belonging to a historically disadvantaged group (eg, ethnic minorities). There was no standardized definition of vulnerability among interviewed experts. If you want to recruit from vulnerable populations (which some experts advise against) the following should also be considered:

- Provide vulnerable people with contact information where they can get help if direct contact cannot be made.
- Vulnerable groups require a higher standard of transparency and consent.

**Post study**

- Publish the study results on the internet to maintain participant engagement and create a sense of ownership.
- Create a poststudy plan if you have created a web-based community.

### Theme 2: Representativeness

#### Increased Bias

Five experts working in social sciences, clinical research, and ethics thought that a sample recruited solely through social media is not representative, especially if the study design requires a high number of participants. Additionally, experts had observed in their studies that certain demographics, for example women, were overrepresented when recruiting through social media, whereas others, for example children, the middle-aged, men, elderly people, and most likely those with less education and income who are and non–English speaking (in an English-speaking country) were underrepresented. A psychologist who recruited for cross-sectional studies summed up their experience as follows:

So, the drawback of Facebook is that... we typically get around 80 percent or even higher who are female. So, we get underrepresentation of males. We get pretty good representation of older adults and younger adults. But in sort of the mid-life, it’s quite hard to obtain people who are in their 30s and 40s, and maybe 50s.

Psychologist

The psychologist then further elaborated that even though Facebook’s business model might be problematic, the potential for research outweighed the platform’s drawbacks:

[Not using social media would be a] missed opportunity... for those marginalized groups of the population who don’t engage in the traditional settings where we do a lot of research, such as in clinical care.... And there’s sort of an ethical question about who you are missing in your research, not just who you are including, but also who you are leaving out.

Psychologist

#### Reduced Bias

One ethicist stated that the question surrounding sample bias depended on the study situation. For studies with very specific target groups that were hard to reach but available on social media, it was more important to find and reach them at all rather than obtaining a representative sample:

And I think you would be creating a bias if you didn’t think with the social media by now, because that is simply the information channel [of the younger generation].

Ethicist

According to our interviewees, many potential participants who rarely responded to traditional recruitment methods were better recruited via social media. Therefore, using social media was considered “the most egalitarian way to do it” by a clinical researcher.

Five experts recruiting for clinical and nonclinical studies were aware that there are different effectiveness levels for recruiting different age groups via social media and that it is more effective with young adults and people older than 50 years while acknowledging that digital literacy becomes an issue for the elderly. An ethicist also noted that ensuring equal access to research is difficult in both the digital and analog world due to the different socioeconomic backgrounds of participants.

While reflecting on their experiences, some experts stated that a multiplatform approach (recruiting via several different social media platforms) and mixed-methods recruitment (using alternative recruitment channels in addition to social media) should be the gold standard as the different recruiting methods complemented each other. Both would help to increase recruitment accrual and reduce the sampling bias. Textbox 1 summarizes practical recommendations on how to increase the reach of the study as phrased by the interviewed experts.

### Theme 3: Web-Based Communities

#### Overview

The experts thought that social media recruitment fits into the modern, fast-communicating world and potential participants’ need for quick responses. The interviewees also observed a new form of debate culture on social networks which harbors its own challenges.

#### Advantages of Web-Based Communities

Experts acknowledged that social media offered the option to establish direct contact between the researcher and participant by establishing closed groups, setting up “fan pages” or directly contacting social media users. Experts noticed several advantages of these web-based communities. First, these communities could increase recruitment effectiveness as social media recruitment could work well for existing patient communities (eg, rare disease communities) who were actively engaging on the internet with each other, as one ethicist reported. Additionally, 2 experts commented that recruiting from existing web-based communities on social media could increase recruitment effectiveness because target populations could be informed more specifically about certain studies. A second advantage mentioned was that answering comments under the post, and as one patient stated, it gives hope to see ongoing research concerning their illness. Moreover, a social scientist said:

maintain[ing] contact with your participants through Facebook and provid[ing] a Facebook page for them... is a good thing to do because it helps them stay in contact with the study, it helps them to feel some ownership of the work that they’re doing with you as participants.

Social scientist

Accordingly, engaging patients in web-based communities was seen as a new dimension of engagement, which was not possible with the classic recruiting methods. For example, patients with the same illness could connect with others if a study was posted in a social media group. One patient also mentioned that as an advantage, as it could give patients reassurance to learn about other patients’ experiences with a certain study.

#### Disadvantages of Web-Based Communities

Despite the previously mentioned potential advantages, many experts acknowledged that engaging and building web-based communities was requiring a lot of personnel (see theme 1) and financial resources. Due to these constraints or data protection regulations, many refrained from engaging in social media community building. Moreover, five experts feared that patients exchanging on social media might lead to the potential unblinding of study participants, thereby undermining the study quality:

That would perhaps be undesirable for the study, because normally you don’t want people, the study participants, to exchange information. And if they suddenly exchange their experiences and who knows, if it is randomised, then it is suddenly no longer randomised, because maybe they all have similar things or conclusions can be drawn. So that’s a problem for the study design as well then.

Social scientist

One ethicist suggested that this could be addressed by educating participants on how randomized studies worked and why it was important that participants do not find out in which study arm they are. Two ethicists added the fear of social pressure to participate in a study if recruitment took place in web-based communities. People may feel more social pressure to participate if they were asked to participate by someone they know. A law expert assumed that such social pressure to participate could potentially compromise the principle of voluntariness for participating in research studies. Additionally, if a web-based community is created for the study participants, a plan needs to be in place on how to progress with this community after the completion of the study (see Textbox 1).

### Theme 4: Privacy

#### Overview

It was noticeable that due to differing legal regulations, perceptions toward privacy implications when using social media for study recruitment differed between European and non-European experts. Some experts considered the different data protection policies, especially between the United States and the European Union, to be the reason for community concerns regarding data protection, that is, less concerns in nations with stricter data protection regulations, that is, the European Union. Yet, some interviewed experts saw the strict interpretation of the GDPR in some member states as a major barrier to social media recruitment.

#### Privacy Is a Big Issue

One expert stated that data protection was important for any study and not specific to social media recruitment. Others disagreed, saying that there were specific risks unique to social media recruitment. For instance, 4 experts noted that from a privacy perspective, inferential targeting and user profiling with algorithms based on artificial intelligence was problematic:

I believe something like this has to be very, very strictly regulated... Because if you use harmless data that people give you - for example, I give my Facebook Likes to Facebook - to derive sensitive data, then you explain to people the moment they provide the harmless data, but not what they derive from it..... So, you’re processing this harmless data for a purpose that you didn’t specify and you’re violating the principle of appropriation. And in the case of instrumental analytics, this has also fallen out of the focus of the highest courts and so totally out of focus... that this happens.

Philosopher

Three clinical and nonclinical experts expressed uncertainty about the implications of the data protection regulations for social media activities, wishing for general legal clarification and ground rules. Consequently, one nonclinical expert advised against setting up project pages or institutional accounts on Facebook to avoid collecting any patient-associated data that would link patients with an illness on social media. One expert also pointed out that the provision of information by potential participants on social media was problematic, even if it was voluntary, as users might not be aware of the consequences, but this was disputed by other experts. Practical recommendations in Textbox 1 address some of these privacy issues, for example, by adding privacy disclaimers to posts on the internet.

#### Privacy Is Not an Issue

One ethicist with no direct web-based recruiting experience saw no problem with placing advertisements on Facebook targeted to a specific audience as long as the message was not coercive. A clinical researcher also thought that this was unproblematic if users deliberately and voluntarily engaged with the advertisement and the study. A social scientist perceived it as unproblematic if people outed themselves on social media as participants because this only revealed them taking part in research but not any personal health information. One ethicist talked about the standard for measuring the risk of social media:

In the United States, the kind of the standard for measuring risk generally is tied to what’s called an “everyday standard”.... So basically, the regulations state that a study is minimal risk and if it’s minimal risk, it’s eligible for all kind of expedited research ethics review and stuff. It’s minimal risk if the risks of the study don’t exceed the risks of everyday life. And so with respect to social media, some people have argued that because social media and just sort of the risks of all these technologies are so much a part of the fabric of our everyday lives now.... These risks seem to be so widely accepted we should count them amongst the risks of everyday life. And if you do that, then at least in the US, it looks like anything involving social media or anything involving one of these tech risks, it’s going to be a minimal risk. It’s going to be minimal risk and not something we should really worry about.

Ethicist

This expert also acknowledged that the “everyday standard” argument was contested among ethicists in the United States and explained why it was a poor argument for not paying more attention to social media risks. Further, they stated that one should be able to scrutinize these everyday risks.

### Practical Recommendations for Using Social Media as a Recruitment Tool

Based on both their experiences and theoretical considerations, the interviewed experts provided practical advice on how to promote a research study via social media (Textbox 1).

## Discussion

### Principal Findings

This qualitative interview study with 30 experts and 6 patients set out to explore the practical benefits and challenges of recruiting study participants with social media in the context of clinical and nonclinical studies. Our findings indicate that there are diverging expert opinions regarding the challenges and benefits of social media recruitment for research studies. Within each presented theme, there were distinct practical benefits and challenges for clinical and nonclinical studies. In the following, we contextualize the expert opinions on social media recruitment and highlight some context-specific benefits and challenges. As discussed elsewhere by our group, the ethical dimension of using social media as a recruitment tool should be evaluated first [14]. This especially applies when vulnerable individuals are recruited, which are here understood as individuals who are not able to protect their own interests [24]. While the GDPR only defines children as vulnerable individuals, other groups, such as the elderly or mentally ill, might also be less able to assess the risks and consequences of the processing of personal data on social media [25]. Overall, there is increasing concern about the use of social media for recruiting vulnerable populations [25].

Contrary to the concerns of the interviewed experts that social media users lack knowledge of data privacy, a recent study suggests that the majority of Americans are aware of the potential risks of web-based data collection [2]. They use social media anyway because they believe that the benefits outweigh the privacy risks and that it is impossible to go through daily life without tracking their data. This is also consistent with the “privacy paradox,” describing the inconsistencies between web-based privacy behavior and attitudes [26]. Studies in the European context showed that for citizens, data protection and data security are very important and they are seriously concerned [27], but even within a country, cultural dimensions affect the level of privacy concerns [28]. Furthermore, our findings show that experts were concerned about the use of commercial social media platforms that use nontransparent, artificial intelligence–based algorithms for inferential targeting and predictive analytics because this might cause serious privacy violations. Recent literature support this point [29,30]. However, for other research studies with healthy patients, some form of presence on the internet, such as a web-based community or a fan page, may help potential study participants to appreciate the research being conducted, especially if study results are later shared in lay language. There is increasing literature on the importance of keeping participants engaged during web-based studies [31-33], but no literature could be found that emphasized the importance of poststudy engagement to create a sense of ownership of study participants.

Many clinical researchers in our study reported that social media recruitment was not cost-effective, which is contrary to what others suggest [34]. This might be because the clinical researchers in our study did not have enough well-trained personnel to deal with the requirement of additional verification steps to be enrolled in a clinical study, as well as with a high number of interested patients signing up via social media. This might have overloaded some study capacities. The nonclinical researchers in our study, most likely accustomed to the high financial and personnel costs of traditional recruitment methods, saw social media as an easier, cheaper, and more efficient way to distribute their web-based surveys, which is in line with other literature on the benefits of social media recruitment for nonclinical studies [9-11,35]. Moving to web-based surveys also increases the geographic reach and increases the speed of recruitment [34].

In addition, some clinicians believed that social media recruitment would not yield representative samples most likely because clinical studies are often not representative due to their very selective nature [36]. Nonclinicians aiming to target a wider population might be confronted with a demographic bias within their recruited sample. This bias can be estimated by comparing the demographic data to estimations of the census bureau of the respective country [9], usually, a national bureau that records demographic data such as age, sex, occupation, and income of its population. In line with what experts mentioned about representativeness, social media were described in some studies to be effective for recruiting younger and older adults within a nonclinical context [10]. This is not in line with official user numbers: in the United States, Facebook is the leading social media site accounting for 64% of all social media site visits [37]. The most prominent age group is aged between 30 and 49 years, and the least prominent group was those aged 65 years or more [38]. Yet, our findings also reflect that in the context of recruiting hard-to-reach populations or rare diseases, it is more important to reach the target population rather than recruiting a representative sample. Thornton et al [11] suggest that in this context, a sample recruited via Facebook is comparable to studies that use traditional recruitment methods. The demographic composition of social media users can vary by country and platform. It is therefore important for researchers to check the country-specific demographic composition of different social media platforms prior to defining which ones to use for study recruitment. This has not only practical but also ethical implications, as it is a question of fairness to include all relevant population groups in research studies, including those that are harder to reach or disadvantaged [14].

Social media recruitment might also help ensure equitable access to research participation, which is known to be very challenging, regardless of web-based or offline recruitment strategies [39]. Many clinical researchers usually have access to venue-based recruitment in the clinical setting, which provides a constant stream of potential study participants. However, if these clinical researchers want to develop a social media recruitment strategy, they must adhere to strict legal frameworks (eg, European Union: GDPR; United States: American Data Privacy and Protection Act) and ethical considerations, such as structured informed consent procedures and privacy requirements, the fundamentals of which are noted in the Declaration of Helsinki. This is complicated by the lack of established guidelines [40-43]. Consequently, some interviewed experts perceived the GDPR as a major barrier to social media recruitment.

### Limitations

Interviews were limited to stakeholders living in Western Europe, the United States, and Australia. Some interview participants had no or little direct practical experience with social media recruitment, particularly members of review boards in the European context. This is most likely because recruitment with social media is comparatively new, and there are no established guidelines. However, these participants contributed with expertise regarding ethical, legal, and social issues related to social media recruitment. While these were mainly theoretical considerations, they are crucial to assess the practical benefits and challenges of social media recruitment. In addition, not all interviewed patients had a firm understanding of how clinical trials are set up but covered a broad spectrum regarding age, digital literacy as well as general attitudes toward social media and data privacy. We did not provide a firm definition of social media to the interview participants and applied their inductive definition to the data analysis. Therefore, the practical guidelines provided in this paper are on a general level but might imply different concrete applications on different social media platforms. Taking a multi-stakeholder approach, we accounted for the variabilities in expertise that are typical for complex, interdisciplinary research questions when analyzing and interpreting the interviews. We aimed to show the diversity of viewpoints of the different stakeholders rather than a quantitative representation of all views.

### Conclusions

We conducted a multi-stakeholder qualitative interview study regarding the benefits and challenges of social media–based recruitment for research studies. Our findings underline the importance of context- and project-specific assessments about the appropriateness and usefulness of social media recruitment before designing the recruitment strategy. For instance, the cost-effectiveness of social media recruitment depends on the context and the design of the research study and the availability and eligibility of alternative recruitment channels. Thus, the place of recruitment (on the internet or offline) is an important factor to consider when designing a recruitment strategy. In many cases, a multiplatform approach (recruiting via several different social media platforms) with mixed-methods recruitment (web-based and offline recruitment channels) might be most beneficial. The different recruiting methods complement each other and help to increase the reach of the study, the recruitment accrual, and the representativeness of the sample. This would help to better meet recruitment targets, which is important for any research study. If a social media–based recruitment strategy is deemed feasible, we suggest consulting the practical expert advice summarized in Textbox 1.

## Appendix

Multimedia Appendix 1 Supplementary material containing detailed participant characteristics, expert interview guide, patient interview guide, list of codes, and author characteristics and contributions.

## Abbreviations

GDPR: General Data Protection Regulation
