# Supplementary material for: Practical Benefits, Challenges, and Recommendations on Social Media Recruitment: Multi-Stakeholder Interview Study
Source: J Med Internet Res. 2023 May 22;25:e44587. doi: 10.2196/44587 (PMC10242465; doi:10.2196/44587)
Supplement: Multimedia Appendix 1 [file jmir_v25i1e44587_app1.docx]

## Participant characteristics

Table 1: Detailed participant characteristics.

| **Area of expertise / Stakeholder group** | **Geographic region** | **Experience with which social media tool** |
| --- | --- | --- |
| Clinical research | Germany | Facebook |
| Clinical research | Germany | Facebook, Twitter |
| Clinical research | Germany | Facebook |
| Clinical research | Germany | n/a |
| Clinical research | Germany | n/a |
| Clinical research | Germany | n/a |
| Clinical research | USA | Facebook |
| Clinical research | USA | Facebook, Instagram, Twitter |
| Clinical research | USA | Facebook |
| Clinical research | Spain | n/a |
| Ethics | Canada | n/a |
| Ethics | USA | Facebook |
| Ethics | USA | Facebook, Craigslist |
| Ethics | USA | n/a |
| Ethics | USA | n/a |
| Ethics | Switzerland | n/a |
| Ethics | Switzerland | n/a |
| Ethics | Germany | n/a |
| Ethics | Germany | n/a |
| Ethics | Germany | n/a |
| Social sciences | Germany | n/a |
| Social sciences | Germany | Facebook, Twitter |
| Social sciences | Australia | Facebook |
| Psychology | Australia | Facebook, Instagram, Twitter |
| Philosophy | Germany | n/a |
| Law | Switzerland | n/a |
| Law | Germany | n/a |
| Law | Germany | n/a |
| Communication (PR) | Germany | Facebook |
| Communication (PR) | Germany | Facebook, Instagram |
| Patient | Germany | n/a |
| Patient | Germany | n/a |
| Patient | Germany | Facebook |
| Patient | Germany | n/a |
| Patient | Germany | n/a |
| Patient | Germany | n/a |
|  |  |  |

## Expert interview guide

### Oral explanation and discussion of the study prior to the interview

As outlined in our study information sheet, I would like to ask you some questions about your views and experiences regarding patient recruitment for clinical trials via social media, especially in connection with the recruitment of vulnerable patient groups, such as the TherVacB cohort. Before we start the interview, I will repeat the most important points from the study information sheet:

This interview study aims to explore the views and experiences of experts on ethical, social, and practical implications of patient recruitment for clinical trials via social media but is not a direct part of any clinical trial. The interview will take about 45 minutes. After that, your participation in this study is completed.

You can end the interview at any time without negative consequences. If you want to end the interview, just let me know. If you wish to withdraw from the study later, you can do so at any time by contacting the study director in writing or by telephone. The contact information is on the information sheet.

The interview is going to be recorded and then transcribed. It will be pseudonymized so that no one can draw any direct conclusions about you. The interview is confidential. We comply with the Bavarian and the European General Data Protection Regulation.

Only the research team has access to the raw data. All data will be saved electronically at the Institute for History and Ethics of Medicine, Technical University Munich, on secure university servers. The encryption remains with the interviewers and is deleted after the end of the project

Do you have any remaining questions regarding the study? *[Clarify all open questions]*

We will register your informed consent for this study in written form. If you consent to participate, please sign the last page of the study information sheet either in print or electronically and send it to us via mail or e-mail. *[Yellow part is obsolete if the interview is held in person – then let the participant sign the informed consent sheet]*

*If informed consent is obtained, announce the start of the recording and start the interview:*

## Interview questions

### Introductory question

- Can you give me an example of when and how you were in touch with patient recruitment for clinical trials via social media?
  - How would you rate this experience?
  - What problems have you experienced?
  - What was particularly important for the successful implementation?
  - Do you know of other examples of clinical trials that recruited participants through social media?
    - If so, please tell me more about your impressions.
  - What challenges do you see before, during, and after a clinical trial regarding patient recruitment via social media?

### Ethical implications

- Can you give me an example of a situation in which you were confronted with (research) ethical problems in the context of patient recruitment for clinical trials via social media? If so, how did you cope with these challenges?
  - What challenges do you see in respecting the autonomy of the patients to be recruited and how would you solve them?
  - What challenges do you see regarding the principle of fairness in recruitment through social media and how would you solve them?
  - What challenges do you see in relation to the harm principle and how would you solve them?
- How do you think patients and the public should be informed about the benefits, but also about the challenges of patient recruitment for clinical trials via social media?
- The patients who are eligible for TherVacB often have particularly vulnerable characteristics (e.g. economic, social). What do you think is special to consider in this context?
- From your point of view, are there any data protection issues that are particularly relevant to patient recruitment for clinical trials via social media?

### Social implications

- What could be the benefits of patient recruitment for clinical trials on social media in public health?
- Do you believe that patient recruitment for clinical trials via social media will lead to cost savings?
- Do you think that all social groups will benefit from patient recruitment for clinical trials via social media?
- How could it be ensured that patient recruitment for clinical trials via social media is fair?

### Legal implications

- What legal problems do you see in connection with patient recruitment for clinical trials via social media?
  - Which data protection aspects must be considered under the GDPR?
- Some argue that private-sector tools and methods, especially those whose algorithms are not open-source (i.e. not publicly available), should not be used for clinical research. What do you think about this?

### Practical implications

- How do you think the research results on patient recruitment for clinical trials via social media find their way into clinical practice? What has worked well so far?
- What are clinical-practical challenges?
- What knowledge and skills should study directors/study recruiters develop to realize the full potential of patient recruitment for clinical trials via social media?
- How do you think this knowledge and skills could be usefully communicated?
- How do you assess the problem that when recruiting patients for clinical studies via social media, potential subjects are addressed on the basis of non-real-world data (i.e. synthetic data such as social media profiles)?
  - How can this problem be dealt with?
  - Have you experienced a situation in which you found dealing with the problem particularly challenging?
  - What are, in your opinion, good solutions to address this issue?

### Closing

Is there anything you'd like to talk about that we haven't discussed?

Thank you very much for your participation!

## Patient interview guide

### Oral explanation and discussion of the study prior to the interview

As outlined in our study information sheet, I would like to ask you some questions about your views and experiences regarding patient recruitment for clinical trials via social media, especially in connection with the recruitment of vulnerable patient groups, such as the TherVacB cohort. Before we start the interview, I will repeat the most important points from the study information sheet:

This interview study aims to explore the views and experiences of experts on ethical, social, and practical implications of patient recruitment for clinical trials via social media but is not a direct part of any clinical trial. The interview will take about 45 minutes. After that, your participation in this study is completed.

You can end the interview at any time without negative consequences. If you want to end the interview, just let me know. If you wish to withdraw from the study later, you can do so at any time by contacting the study director in writing or by telephone. The contact information is on the information sheet.

The interview is going to be recorded and then transcribed. It will be pseudonymized so that no one can draw any direct conclusions about you. The interview is confidential. We comply with the Bavarian and the European General Data Protection Regulation.

Only the research team has access to the raw data. All data will be saved electronically at the Institute for History and Ethics of Medicine, Technical University Munich, on secure university servers. The encryption remains with the interviewers and is deleted after the end of the project

Do you have any remaining questions regarding the study? *[Clarify all open questions]*

We will register your informed consent for this study in written form. If you consent to participate, please sign the last page of the study information sheet either in print or electronically and send it to us via mail or e-mail. *[Yellow part is obsolete if the interview is held in person – then let the participant sign the informed consent sheet]*

*If informed consent is obtained, announce the start of the recording and start the interview:*

### Interview questions

### Introductory question

- What do you use social media for?

### General attitude and values

- What is your general attitude towards social media?
- Are you active in social media regarding your disease? (e.g. in appropriate Facebook groups, own posts on the topic, linking to matching posts from others, friends/follows of other hepatitis B sufferers)
  - How could you imagine being recruited via social media for a clinical hepatitis B study?
  - What advantages and disadvantages do you see?

### Expectations

- What would/is particularly important to you when being recruited for a clinical trial?
- What information do you need to feel comfortable when being recruited through social media?

### Worries

- Have you ever had negative experiences on social media?
  - If so, what kind? How does this affect potential recruitment for clinical trials?
  - Do you have data protection concerns? How could these be resolved from your point of view?
- Can you remember situations in which you or others affected by the same illness as you have suffered disadvantages due to your illness?
  - How could the inclusion of social media highlight such disadvantages?
  - How could any risks of recruitment via social media be reduced or prevented?

### Closing

Is there anything you'd like to talk about that we haven't discussed?

Thank you very much for your participation!

## List of codes

| **Code groups** | **Codes** |
| --- | --- |
| Benefits of SMR | Benefits of SMR |
| Ethics | Ethics_Cause harm / pain / disturbances / distress |
|  | Ethics_Data protection /privacy issues |
|  | Ethics_Discrimination / stigma |
|  | Ethics_Generalizability, selection bias, fairness |
|  | Ethics_growing awareness of ethical issues |
|  | Ethics_Transparency |
|  | Ethics_Vulnerable populations |
| Good research ethics | Research ethics_conflicts of interest |
|  | Research ethics_quality assessment |
| Legal | Legal_Regulatory issues |
| Patients' perspectives | Experts about patients acceptance of SMR |
|  | Patients_acceptance of SMR |
|  | Patients_experiences with SMR |
|  | Patients_social media literacy |
|  | Patients_therapeutic misconceptions |
|  | Patients_usage habits |
| Practical implications | Other practical challenges |
|  | Potential solutions for practical SMR problems |
|  | Resources needed to conduct SMR |
|  | SMR Effectiveness |
|  | SMR professionalization |
|  | SMR strategy development |
| SMR Particularities | Justifications pro/contra SMR |
|  | Particularities_clinical trials vs other research |
|  | Particularities_Europe/America |
|  | Particularities_SMR compared to other recruitment methods |

## Author characteristics and contributions

Nina Goldman, Ph.D., female, postdoctoral researcher, trained in social geography.

Theresa Willem, M.A., female, Ph.D. candidate, trained in media sciences including qualitative research.

Alena Buyx, Dr med, full professor, trained in biomedical ethics including qualitative research.

Bettina M. Zimmermann, Ph.D., female, postdoctoral researcher, trained in empirical bioethics including qualitative research.

### Potential biases:

All authors hold social media accounts themselves and thus have personal positive or negative experiences with its use. None of the authors had used social media to recruit for research studies when conducting this study.
